# The vineyard as a therapeutic landscape of the mind: preliminary results of a pilot study

**DOI:** 10.1192/j.eurpsy.2023.1289

**Published:** 2023-07-19

**Authors:** E. Rossero, A. Barbieri

**Affiliations:** 1 Eclectica+ Research and Training, Turin; 2 Mental Health Department, ASL CN1, Cuneo, Italy

## Abstract

**Introduction:**

Young people represent a vulnerable population, with 75% of mental disorders first emerging before 25 years of age. This pilot stems from the acknowledged need to design and test non-stigmatizing programs that are appealing to young people and suited for the protean mental health problems that they experience.

**Objectives:**

The study involves a group of youths (aged 16-25) with different forms of mental ill-health in a locally and culturally meaningful activity, namely hand-harvesting grape in the renowned area of Langhe (Italy). The aim is to investigate viticultural practices as possibly effective in supporting recovery by promoting social interaction and fostering a sense of belonging in the broader process of winemaking.

**Methods:**

The project is multidisciplinary in its design and implementation, involving psychiatrists, psychologists, rehabilitation specialists and sociologists. Research methods include clinical assessment, participant observation, and semi-structured interviews with the participants.

**Results:**

During the harvest season, a stable group of participants has been involved in a one-to-one relationship with professional vine growers. This relational geometry was built around the performance of a practical task: that of filling in a box with manually harvested grape and moving it along the rows of vines. Within each dyad, which represents the most fragile and intimate of all social forms, practical knowledge has been conveyed from the experienced worker to the youth. Most importantly, the repeated encounters provided an opportunity for human interaction and exchange that went beyond the activity being performed, involving the gradual disclosure of self, the ability to listen, connect and empathize with personal stories from diverse backgrounds. Participants’ narratives collected during and after the pilot describe the vineyard as a psychic more than a physical place – a landscape of the mind, structured around the emotional and sensorial contents of the experience. The study’s core finding emerging from fieldwork and youths’ accounts is the beneficial effects of the intervention on transdiagnostic factors such as social anxiety symptoms, low self-efficacy and poor social skills.

**Conclusions:**

The pilot provides suggestions to orient meaningful and non-stigmatising programs for vulnerable young people, hosted in landscapes that can become therapeutic not by virtue of their aesthetic features, but because of the access they provide to social (i.e. opportunities for new relationships), material (occasions to create and share something tangible) and affective (promotion of positive emotions, containment of loneliness and feelings of inadequacy) resources.

**Disclosure of Interest:**

None Declared

